# Analysis of lower limb muscle strength characteristics of amateur runners with patellofemoral pain: A cross-sectional study

**DOI:** 10.1371/journal.pone.0305141

**Published:** 2024-06-10

**Authors:** Gangrui Chen, Zhongke Gu, Peixun Wang, Yuxuan Qi, Jiansong Dai

**Affiliations:** 1 Learning and Training Integration Center, Nanjing Sport Institute, Nanjing, China; 2 Department of Sports and Health Science, Nanjing Sport Institute, Nanjing, China; 3 School of Physical Education, Performance and Sport Leadership, Springfield College, Springfield, Massachusetts, United States of America; The Wingate College of Physical Education and Sports Sciences at the Wingate Institute, IL, ISRAEL

## Abstract

To analyze the lower limb muscle strength characteristics of amateur runners with patellofemoral pain (PFP). Sixty amateur runners (30 in the knee pain group, 30 in the control group) underwent measurements of hip abduction strength, knee extension strength, and knee flexion strength. Additionally, the hamstring/quadriceps ratio and limb strength symmetry index were calculated for all participants. Statistical analyses were conducted using linear mixed models. The hip abduction and knee extensors strength of amateur runners with PFP was significantly lower than that of the control group. The hamstring/quadriceps ratio was significantly higher in the male knee pain group compared to the control group, while no significant difference was found between the female knee pain group and the control group. Furthermore, both the hip abduction strength symmetry index and knee extensors symmetry index were significantly lower in the knee pain group compared to the control group. Amateur runners with PFP exhibit distinct lower limb strength characteristics compared to non-knee pain runners. Additionally, differences in lower limb strength characteristics between male and female amateur runners with PFP were observed. These findings emphasize the importance of considering functional and gender differences in PFP rehabilitation training.

## Introduction

Patellofemoral pain (PFP) is a commonly non-traumatic chronic injury with the main symptom of diffuse anterior knee pain when the joint is loaded (e.g., squatting, running, stair climbing) [1]. PFP is commonly observed in physically active populations, such as amateur enthusiasts and elite athletes, with an annual incidence rate of 4%-21% among runners. The incidence rate of PFP is as high as 25% in female runners, which is twice that of male runners [2]. However, the number of female runners was only half that of male runners [3]. Moreover, PFP is characterized by persistent symptoms and high recurrence rates, with over one-third of patients still experiencing symptoms after 12 months of rehabilitation treatment, which can have an impact on lower limb function [4].

Typically, patellofemoral pain is caused by altered stress on the patellofemoral joint from intrinsic knee factors, alterations in the kinetic chain, or errors in training [5]. Existing research has demonstrated a strong correlation between PFP and deficits in quadriceps and hip abduction strength [6, 7]. Insufficient hip abduction muscle strength can lead to functional impairment of lower limb joint alignment, increasing patellofemoral joint stress and thereby elevating the risk of injury [8]. Therefore, strengthening exercises for the quadriceps and hip abduction muscles are commonly used as a treatment for PFP in clinical rehabilitation [9]. However, studies have found that approximately 50% of PFP patients still experience knee pain within five to eight years after local muscle strengthening training [10]. Therefore, an analysis of the lower limb muscle strength characteristics of PFP patients is of great significance for a deeper understanding of the prevention and rehabilitation of this injury and for improving long-term treatment outcomes.

Bilateral muscle asymmetry and antagonistic muscle imbalance are also considered contributing factors to various injuries [11]. Sapega et al. proposed that 15–20% difference in bilateral strength is considered as bilateral asymmetry, and difference in lower limb strength of more than 20% is associated with an increased risk of athlete injury [12]. The ratio of hamstring to quadriceps concentric strength (H:Q) is not only used to test the muscle balance between quadriceps and hamstring muscles, but also to evaluate knee joint functional capacity. This ratio, ranging from 0.5 to 0.8, is considered the relatively ideal range for injury prevention and rehabilitation [13]. At present, although there are more studies on lower limb muscle strength in PFP patients, there are fewer studies on limb symmetry index (LSI) and antagonist muscle balance in PFP amateur runners. Whether there are obvious lower limb muscle strength asymmetries and imbalances in PFP amateur runners, and whether there are differences in lower limb symmetry and balance in PFP amateur runners of different genders require in-depth studies on the characteristics of lower limb muscle strength in PFP amateur runners and the gender differences, so that the rehabilitation treatment of PFP patients should be functional and gender-specific.

This investigate aims to deepen the understanding of lower limb muscle strength in amateur runners with PFP by analyzing the differences in lower limb isokinetic muscle strength between male and female PFP and non-PFP runners. This study hypothesized that lower limb muscle strength characteristics and limb symmetry index of amateur runners with PFP would differ from those of amateur runners without injury.

## Materials and methods

### Participants

G-Power software (G*Power©, University of Dusseldorf, Germany) was used to estimate the sample size according to a recent similar study that assessed knee extensors and hip abductors [14, 15]. The analysis revealed that to perform fixed effects, main effects and interactions Analysis of variance (ANOVA) with an effect size of 0.50, power of 0.80, and alpha of < 0.05, we would need at least 9 participants. we determined a minimum sample size of 60 participants was required to be assigned into four groups: 20 with male knee pain, 20 with male control, 10 with female knee pain, 10 with female control. Recruitment of amateur runners in the Nanjing area was conducted through advertising on social media platforms.

The recruitment period for participants in this study is from July 5, 2021 to August 10, 2021. Thirty amateur runners (aged 18–50) with patellofemoral joint pain were selected as the knee pain group, consisting of 20 males and 10 females, all experiencing pain in the right knee. Thirty injury-free amateur runners were matched to the knee pain group based on age, height, weight, and gender, forming the control group. On the day of testing, none of the participants exhibited acute knee pain symptoms, and they were provided with an explanation of the research purpose. This study was conducted at the Physical Training and Rehabilitation Laboratory, Nanjing Sport Institute, and was approved by the ethics committee of the Nanjing Sport Institute (No.: RT-2020-3). Table 1 displays the physical characteristics of the participants included in this investigation.

**Table 1:** Characteristics of the participants.

	Male			Female		
	Knee pain (n = 20)	Control (n = 20)	P	Knee Pain (n = 10)	Control (n = 10)	P
Age(y)	30.5±4.12	28.7±3.13	0.118	30.6±5.66	27.9±4.56	0.255
Height(cm)	171.95±7.57	171.45±6.96	0.829	163.00±6.11	165.1±4.35	0.144
Weight(kg)	66.40±11.21	65.80±10.41	0.861	55.90±5.97	58.60±5.16	0.153
BMI	22.35±2.64	22.26±2.44	0.915	21.01±1.56	21.54±1.65	0.470
PFP Affected Side	Right			Right		

All runners had a running frequency of at least three times per week and a single run distance exceeding 3 kilometers over the past three months [16, 17]. The screening of subjects was uniformly carried out by a rehabilitation therapist, following the diagnostic criteria for PFP proposed by Resnick [18]. The inclusion criteria were: (1) obvious pain upon palpation of the medial and lateral patella and its periphery; (2) symptoms of "giving way" or peripatellar pain, accompanied by pain when going up or down stairs and squatting, affecting normal actions such as running and jumping; (3) positive patellar grind test, resistive knee extension test, and single leg squat test; The exclusion criteria were: (1) acute phase of inflammation, meniscal injuries, ligamentous injuries of the knee, intra-articular fractures, bone tumors, bone tuberculosis, and neurovascular injuries; (2) subjects uncooperative or with serious systemic diseases such as cardiac, hepatic, renal, or hematological disorders; (3) intra-articular injection of hyaluronic acid or corticosteroids in the past six months.

### Data collection

#### Knee extension and flexion and hip abduction strength tests

The BTE PrimusRS simulation test and training system (BTE, Hanover, MD, USA) was utilized to measure the hip abductor strength, Knee extension and flexion strength of the participants’ lower limbs. The primary measurement in this study was Peak Torque (PT), and the maximum peak force was normalized to body weight to mitigate the influence of weight. The unit of measurement is Nm/kg. All tests were performed using concentric contractions throughout the full range of joint motion. The knee extension and flexion strength tests were conducted at angular velocities of 30°/s, 60°/s, and 120°/s. Hip abduction strength was assessed at angular velocities of 30°/s and 60°/s. Three trials were performed at each angular velocity, and the maximum value obtained from these trials was used for statistical analysis.

#### LSI

Limb symmetry index was calculated in all unilateral tests performed (LSI = worse leg/better leg × 100) [19, 20].

#### Hamstring to quadriceps ratio

Hamstring peak concentric torque/quadriceps peak concentric torque.

### Statistical analysis

The study’s statistical analysis was performed using SAS JMP 17.0 software with a level of significance of p < 0.05. A linear mixed model was used for statistical analysis, with group (knee pain group and control group), gender (male and female), lower limb (left side, right side) were analyzed as fixed factors for hip abduction strength, knee extension strength, knee flexion strength, and H:Q ratio, and group (knee pain group and control group), gender (male and female) were analyzed as fixed factors for hip abduction strength symmetry index, knee extension strength symmetry index, and knee flexion strength symmetry index, and if there was an interaction, the Tukeys multiple comparison test was used for post hoc multiple comparisons, significant main effects were reported if there were no significant interactions. Effect sizes were calculated using partial η squared (ηp2) and interpreted as small (0.01), medium (0.06), or large (0.14).

## Results

### Anthropometric measurements

There was no significant difference between the male height, weight, age, and BMI knee pain group and the control group (P = 0.118, P = 0.829 P = 0.861, P = 0.915), and no significant difference between the female height, weight, age, and BMI knee pain group and the control group (P = 0.255, P = 0.144 P = 0.153, P = 0.470). The results are presented in Table 1.

### Comparison of peak torque in different parts of lower limbs

The interaction effects of group, gender, and limb side on hip abduction strength at angular velocities of 30°/s and 60°/s were not significant (P = 0.902, ηp^2^ = 0.000; P = 0.527, ηp^2^ = 0.003). However, the interaction effect of group and limb side at 30°/s and 60°/s angular velocities was significant (P = 0.046, ηp^2^ = 0.103; P = 0.046, ηp^2^ = 0.092), with the right side of the knee pain group significantly lower than the right side of the control group (P = 0.011, P = 0.003). For knee extension strength at angular velocities of 30°/s, 60°/s, and 120°/s, the interaction effects of group, gender, and limb side were not significant (P = 0.997, ηp^2^ = 0.000; P = 0.366, ηp^2^ = 0.007; P = 0.853, ηp^2^ = 0.000;). Likewise, the interaction effects of group-gender (P = 0.242, ηp^2^ = 0.001; P = 0.262, ηp^2^ = 0.011; P = 0.373, ηp^2^ = 0.006), group-limb side (P = 0.855, ηp^2^ = 0.000; P = 0.585, ηp^2^ = 0.003; P = 0.267, ηp^2^ = 0.010), and gender-limb side (P = 0.628, η_p_^2^ = 0.002; P = 0.759, η_p_^2^ = 0.001; P = 0.833, η_p_^2^ = 0.000) were not significant, the knee pain group was significantly lower than the control group at 120°/s (p = 0.012, ηp2 = 0.151). Similarly, for knee flexion strength at angular velocities of 30°/s, 60°/s, and 120°/s, the interaction effects of group, gender, and limb side were not significant (P = 0.893, ηp^2^ = 0.000; P = 0.980, ηp^2^ = 0.000; P = 0.995, ηp^2^ = 0.000). Additionally, the interaction effects of group-gender (P = 0.402, ηp^2^ = 0.006; P = 0.832, ηp^2^ = 0.000; P = 0.801, ηp^2^ = 0.001), group-limb side (P = 0.654, ηp^2^ = 0.002; P = 0.878, ηp^2^ = 0.000; P = 0.446, ηp^2^ = 0.005), and gender-limb side (P = 0.254, ηp^2^ = 0.011; P = 0.979, ηp^2^ = 0.000; P = 0.446, ηp^2^ = 0.005) were not significant. The results are presented in Table 2.

**Table 2:** Comparison of peak torque in different parts of lower limbs (Nm/kg).

Parts	AV(°/s)	Male				Female			
		Knee pain		Control		Knee pain		Control	
		Left	Right	Left	Right	Left	Right	Left	Right
Hip Abduction	30	2.79±1.01	2.21±1.02*	2.72±0.81	2.85±0.7	2.66±0.66	2.11±0.7*	2.87±0.81	2.95±0.97
	60	2.69±0.84	2.18±0.88*	2.99±0.8	2.88±0.66	2.42±0.61	1.89±0.59*	2.68±0.67	2.92±0.72
Knee Extensors	30	4.9±1.46	4.55±1.8	5.4±0.89	4.98±1.03	5.31±1.32	5.2±1.31	5.22±0.9	5.04±1.14
	60	4.58±1.58	4.21±1.75	4.95±1.07	5.34±1.04	4.56±0.98	4.83±1.41	4.83±1.22	4.9±1.1
	120	3.76±1.51*	3.39±1.28*	4.26±1.18	4.5±1.06	4.31±1.04*	3.93±1.02*	4.48±1.28	4.53±0.99
Knee Flexors	30	3.54±1.26	3.69±1.66	3.65±0.94	3.67±0.72	3.63±0.96	3.35±1.04	3.45±0.75	2.92±0.67
	60	3.64±1.08	3.73±1.62	3.52±0.77	3.55±0.77	3.32±0.73	3.4±1.07	3.28±0.67	3.3±0.52
	120	3.02±1.12	3.44±1.3	3.15±0.92	3.29±0.88	2.97±0.73	3.11±0.66	3.19±0.88	3.04±0.55

AV: Angular velocity * Compared to control group, # Compared to males, & Compared to left.

### Hamstring to quadriceps ratio in runners with knee pain and control

**Fig 1:**
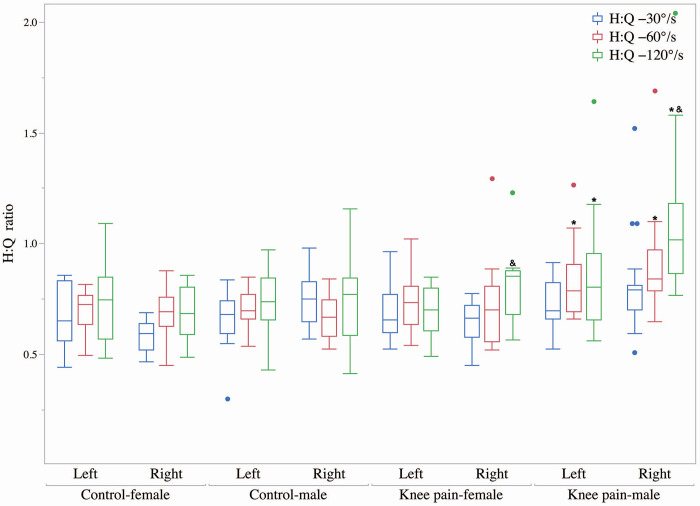
Hamstring to quadriceps ratio. * Compared to control group, # Compared to males, & Compared to left.

The interaction effects of group, gender, and limb side on the H:Q ratio at angular velocities of 30°/s, 60°/s, and 120°/s were not significant (P = 0.867, ηp^2^ = 0.000; P = 0.338, ηp^2^ = 0.006; P = 0.762, ηp^2^ = 0.001). However, the interaction effect of group and gender on the H:Q ratio at angular velocities of 60°/s and 120°/s was significant (P = 0.025, ηp^2^ = 0.085; P = 0.04, ηp^2^ = 0.126). Specifically, the male knee pain group exhibited a significantly higher H:Q ratio compared to the control group at 60°/s (P = 0.001) and 120°/s (P = 0.001), while there was no significant difference between the female knee pain group and the control group at 60°/s (P = 0.831) and 120°/s (P = 0.902). At an angular velocity of 120°/s, the interaction effect of group and limb side on the H:Q ratio was significant (P = 0.017, ηp^2^ = 0.098;). The right side of the knee pain group showed a significantly higher H:Q ratio compared to the left side of the knee pain group and the right side of the control group (P = 0.019, P = 0.003), as illustrated in Fig 1.

### Comparison of lower limb strength LSI in runners with knee pain and control

**Fig 2:**
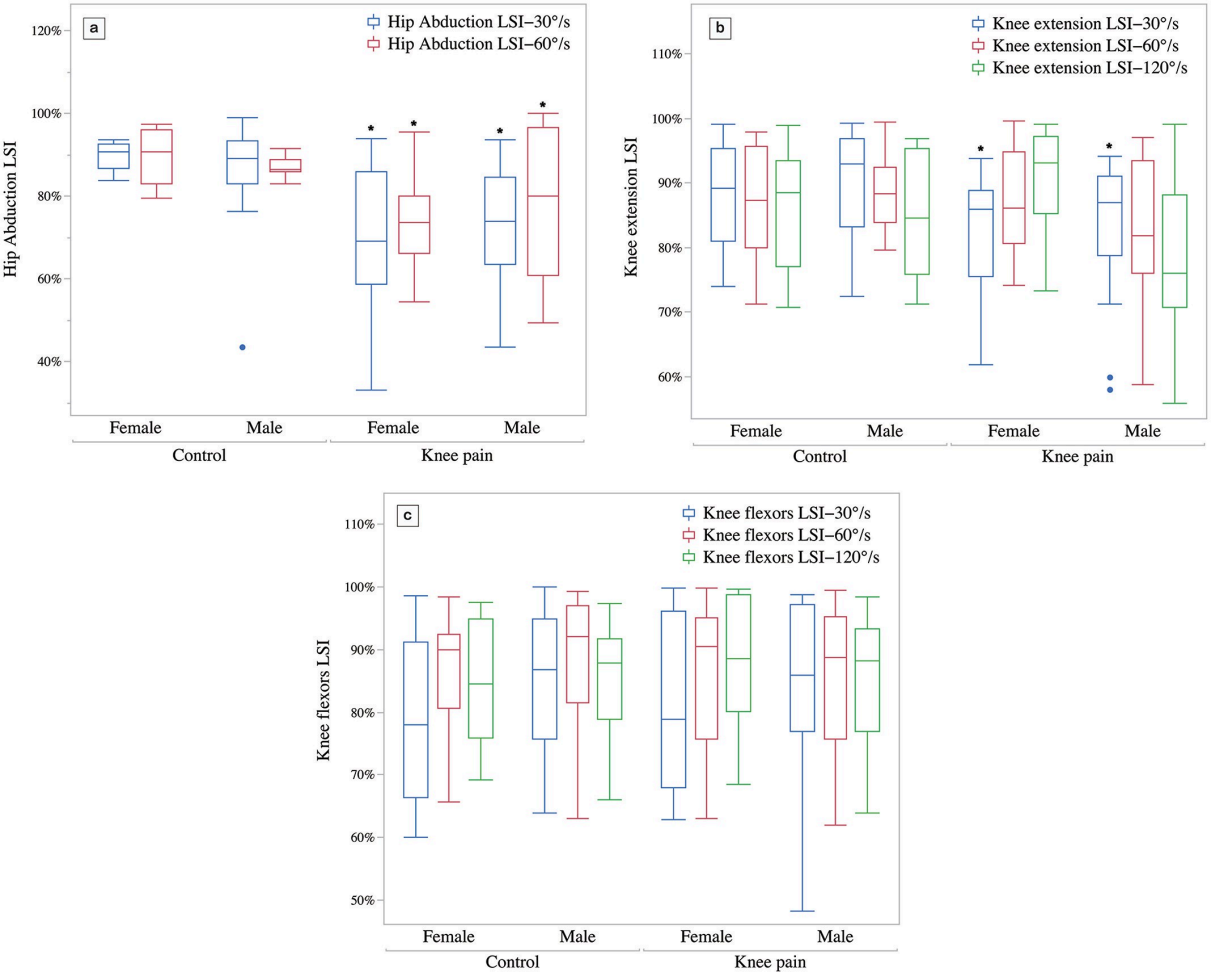
Lower limb strength LSI (%). a) Hip abduction LSI, b) Knee extension LSI, c) Knee flexors LSI. * Compared to control group, # Compared to males.

The interaction of group and sex on the LSI of hip abduction strength at 30°/s and 60°/s angular velocity was not significant (P = 0.679, ηp^2^ = 0.005; P = 0.428 ηp^2^ = 0.006;). The knee pain group was significantly lower than the control group (P = 0.001, ηp^2^ = 0.241 at 30°/s, P = 0.002, ηp^2^ = 0.164 at 60°/s), but there was no significant difference between males and females (P = 0.760, ηp^2^ = 0.001 at 30°/s, P = 0.985, ηp^2^ = 0.000 at 60°/s). There was no significant interaction effect of group and sex on the LSI of knee extension force at 30°/s (P = 0.963, ηp^2^ = 0.000), which was significantly lower in the knee pain group than in the control group (P = 0.016, ηp^2^ = 0.097), as illustrated in Fig 2.

## Discussion

This study partially supports the hypothesis that there exist differences in lower limb muscle strength characteristics between amateur runners with PFP and those without knee pain. Relative to the control group, PFP amateur runners exhibited reduced hip abduction and extensors strength and its LSI, increased H:Q. These results suggest that PFP impacts lower limb local muscle strength, LSI, H:Q, and other muscle strength characteristics in amateur runners, and that the characteristics of lower limb muscle strength are not consistent among PFP amateur runners of different genders.

This study found that the right hip abductor strength of amateur runners with PFP was significantly lower than that of the control group runners on the right side (P < 0.05), indicating that weak hip abductor strength is one of the characteristics of PFP runners, consistent with previous research findings. Culvenor et al. found that the hip abduction and external rotation strength of young women with PFP was significantly lower than their healthy counterparts [21]. Rathleff et al. proposed that the lack of hip abduction and external rotation strength could lead to abnormal patellofemoral joint force lines and cause PFP [22]. Alammari et al. found that specialized gluteus medius strength training for six weeks significantly reduced pain in more than 90% of long-distance runners with PFP [9].

The main muscles involved in hip abduction are the gluteus medius, gluteus minimus, and piriformis, with the gluteus medius and minimus playing a more significant role. The functions of the gluteus medius and minimus are essentially identical: apart from enabling hip abduction, the anterior fascicle can induce internal rotation of the hip joint, while the posterior fascicle enables external rotation. Their primary function is similar to that of the rotator cuff muscle group, serving as stabilizers of the hip joint. Since running is a single-leg support exercise, weakened hip abduction strength can lead to greater knee varus and hip adduction and internal rotation during the ground support phase of running. The internal rotation of the femur can significantly increase the lateral pressure of the patella, which is a critical mechanism leading to abnormal patella movement and the onset of patellofemoral joint pain during long-term continuous running.

The H:Q ratio is a common metric used in the evaluation of muscular strength, reflecting the balance and coordination between the primary mover and antagonist’s muscles of the knee joint. International studies suggest that a normal H:Q ratio falls between 30% to 90%, while domestic research proposes a 50% to 60% range considering racial differences among Asians. However, the majority of studies still maintain that the optimal H:Q ratio lies between 50% to 80%, where an overly low (<50%) or excessively high (>80%) ratio could potentially precipitate knee joint dysfunction.

The H:Q ratio is a widely employed measure for assessing muscle strength, reflecting the balance and coordination between the agonist and antagonist muscle groups of the knee joint [13]. Most studies indicate that the optimal range for the H:Q ratio is between 50% and 80%. Values below 50% or above 80% are considered to potentially indicate aberrations in knee joint functionality [23]. This study observed that the H:Q ratio at an angular speed of 60°/s on the left side, as well as 60°/s and 120°/s on the right side, was significantly higher in male amateur runners with PFP than those without knee pain (P<0.05). This is consistent with previous research suggesting a higher H:Q ratio on the affected side compared to the healthy side in PFP cases [13]. This outcome could be attributed to the fact that, although the hamstring strength of male amateur runners with PFP did not significantly differ from the control group, their quadriceps strength was significantly lower, thus resulting in a higher H:Q ratio. Therefore, based on the study, we infer that when the H:Q ratio exceeds 80%, the reduction in quadriceps strength could be the primary cause of PFP. When the ratio falls below 50%, it could be inferred that significantly lower hamstring strength relative to the quadriceps could be the principal cause of PFP. Both overly high and low H:Q ratios can lead to an imbalance in the knee flexor-extensor muscle group, consequently increasing patellofemoral pressure and inducing PFP. In this study, the male knee pain group exhibited an H:Q ratio above 80% at an angular speed of 120°/s on both sides, whereas the control group showed an H:Q ratio between 60% to 80% at all speeds on both sides. A similar pattern was observed in female groups, with the female knee pain group presenting H:Q ratios above 80%, which were not found in the control group. These results indicate that amateur runners with PFP demonstrate elevated H:Q ratios.

The LSI is considered an effective tool for monitoring injury risk in athletes and evaluating whether they can return to the field after ACL injury [19]. To meet the standard for returning to the field after ACL surgery, the LSI of quadriceps and hamstring strength, single-leg jump, and other indicators should be between 85% and 90% [24, 25]. The results of this study showed that the LSI of hip abduction strength in female PFP amateur runners and the LSI of hip abduction and knee extensors strength in male PFP amateur runners were lower than those in the non-PFP group, and the LSI of male and female PFP runners were less than 85%. This indicates that amateur runners with PFP have significant lower limb strength asymmetry. The LSI of lower limb can be used to predict and evaluate the risk of patellofemoral joint pain, and it is recommended that the LSI value of 85% be used as the threshold for PFP injury.

In summary, this study found that male patients exhibited significant decreases in hip abduction strength and LSI, quadriceps strength and LSI, and significant increases in H:Q ratio, while female patients only exhibited significant decreases in hip abduction strength and symmetry index. Therefore, in the clinical treatment and rehabilitation training of PFP patients of different genders, attention should be paid to the rehabilitation focus. Strengthening hip abduction strength is a basic measure, but male patients should also pay attention to strengthening quadriceps training. Although Khayambashi et al. suggested that isolated quadriceps and hip abduction muscle training can effectively treat PFP [26], Na et al. found that simple hip strengthening can also help reduce pain in PFP patients [27], but more targeted, diverse, and balanced compound muscle training may be more helpful in reducing knee pain. It is suggested that future studies can propose rehabilitation training strategies for PFP based on gender characteristics, such as whether the combination of quadriceps and hip abduction strength training has a better therapeutic effect on the pain level, lower limb function, and running biomechanical characteristics of PFP patients.

## Limitations

This study was a cross-sectional study and failed to clarify the causal relationship between PFP and lower limb muscle strength characteristics. It is recommended that a prospective cohort study of amateur runners and a rehabilitation training intervention study for amateur runners with PFP be conducted at a later stage to further clarify the causal relationship between PFP and lower limb muscle strength characteristics.

## Conclusions

The amateur PFP runners demonstrated weaker hip abduction and knee extensors strength, lower hip abduction LSI and knee extensors LSI. Male PFP runners also exhibited higher H:Q ratio. Although the hamstring muscle strength and limb symmetry index of PFP runners were lower than control runners, there was no significant difference between the knee pain and control groups. Therefore, hamstring muscle strength may not be considered a typical lower limb strength characteristic of amateur PFP runners.

## Supporting information

S1 File Supporting primary data. (XLSX)
